# Seroprevalence and risk factors for *Toxoplasma gondii* infection in solid organ transplant patients: A global systematic review and meta-analysis

**DOI:** 10.1016/j.parepi.2025.e00421

**Published:** 2025-03-07

**Authors:** Mina Mamizadeh, Farajolah Maleki, Mohammad Reza Mohammadi, Laya Shamsi, Ali Asghari, Ali Pouryousef

**Affiliations:** a Department of Dermatology, School of Medicine, Ilam University of Medical Sciences, Ilam, Iran; b Zoonotic Diseases Research Center, Ilam University of Medical Sciences, Ilam, Iran; c Clinical Research Development Unit, Shahid Mostafa Khomeini Hospital, Ilam University of Medical Sciences, Ilam, Iran; d Department of Bacteriology, Faculty of Medical Sciences, Tarbiat Modares University, Tehran, Iran; e Department of Pathobiology, Faculty of Veterinary Medicine, Urmia University, Urmia, Iran; f Medical Microbiology Research Center, Qazvin University of Medical Sciences, Qazvin, Iran; g Leishmaniasis Research Center, Sabzevar University of Medical Sciences, Sabzevar, Iran

**Keywords:** *Toxoplasma gondii*, Seroprevalence, Odds ratio, Solid organ, Transplant recipients

## Abstract

This study aimed to assess the global seroprevalence of IgG and IgM antibodies against *Toxoplasma gondii* (*T. gondii*) in solid organ transplant (SOT) recipients (kidney, liver, heart) through a literature review of studies published until October 24, 2024. Selected studies reported data on anti-*T. gondii* IgG and IgM seroprevalence in the post-transplant stage of SOT recipients. A random-effects model estimated pooled seroprevalence rates, and heterogeneity was evaluated using the I^2^ statistic. Sensitivity analysis examined prevalence changes after excluding studies, while subgroup analysis of IgG seroprevalence accounted for publication years, countries, continents, WHO regions, sample sizes, and types of transplanted organs. Out of 26 articles and 29 datasets analyzed, 21 articles and 24 datasets involving 19,391 transplant recipients and 880 controls were used to assess anti-*T. gondii* IgG and IgM seroprevalence and odds ratios (ORs). Additionally, 8 articles reported the anti-*T. gondii* IgG serostatus of donors and recipients. The pooled IgG seropositivity for *T. gondii* in SOT recipients was 9.8 % (95 % CI, 4.7–19.4 %), showing significant variation by region and organ type. The anti-*T. gondii* IgM seroprevalence in SOT recipients was 6.4 % (95 % CI, 3.3–12 %). Renal transplant recipients exhibited higher IgG seroprevalence compared to liver and heart transplant recipients. The pooled OR for *T. gondii* infections in SOT recipients vs. controls was 1.39 (95 % CI, 0.95–2.04, *P* = 0.08). The highest pooled anti-*T. gondii* IgG serostatus was 50.7 % in the undetermined group, followed by 38 % in the D−/R- group, 15.4 % in the D−/R+ group, 10.6 % in the D+/R- group, and 9.9 % in the D+/R+ group. Overall, *T. gondii* active infections and its increased risk trend in SOT recipients should not be overlooked.

## 1 Introduction

*Toxoplasma gondii* is a significant obligate intracellular protozoan responsible for the opportunistic disease toxoplasmosis (Adem and Ame, 2023). Its global prevalence is estimated at 25.7 % (Molan et al., 2019). The infection is primarily transmitted through contaminated food or water containing sporulated oocysts or by consuming undercooked infected meat (Shapiro et al., 2019). Other less recognized transmission routes include congenital transmission, blood transfusions, and organ transplantation (Pinto-Ferreira et al., 2019; Shapiro et al., 2019).

Healthy individuals infected with *T. gondii* typically remain asymptomatic, but those with immunodeficiency, such as HIV/AIDS patients, transplant recipients, cancer patients, and others undergoing prolonged immunosuppressive therapy, are at risk of severe, life-threatening infections (Teimouri et al., 2022; Wesołowski et al., 2023). Latent toxoplasmosis is common among COVID-19 patients and may suggest an increased risk of infection (Hamdy et al., 2024). A previous study confirmed the unequivocally circulating of *T. gondii* type I in these patients, but no significant link was found between *T. gondii* infection and COVID-19 severity (Hasanzadeh et al., 2024). *T. gondii* represents a considerable risk for cancer patients undergoing chemotherapy. Research highlights the necessity for regular screening, diagnosis, and treatment of *T. gondii* infections, as this poses a significant health issue for this vulnerable group (Ali et al., 2019).

Febrile myocarditis, encephalitis, and pneumonitis are the most common clinical manifestations of *T. gondii* infection in post-transplant patients (Derouin et al., 2008; Kucerova and Cervinkova, 2016). The risk of reactivating dormant tissue cysts is heightened in solid organ transplant recipients due to immunosuppressive therapy or a mismatch between seropositive donors and seronegative recipients (Khurana and Batra, 2016). The likelihood of infection in SOT recipients varies based on the infection dose, duration of immunosuppressive medication, and type of grafted tissue (Dard et al., 2018). While heart transplant recipients face the highest risk, other organs such as the liver, kidney, intestine, pancreas, and bone marrow are also involved in infection transmission (Coster, 2013; Robert-Gangneux et al., 2018).

Given the rising number of SOT patients globally, screening tests for graft donors and recipients are essential to identify those at high risk for *T. gondii* infections (Pagalilauan and Limaye, 2013). Numerous studies have examined the prevalence of *T. gondii* in SOT patients, but a comprehensive analysis is needed for a better understanding of the infection risk. Therefore, we designed a systematic review and meta-analysis to assess the seroprevalence of *T. gondii* infection in post-transplant stage of SOT recipients.

## 2 Methods

### 2.1 Study design

This systematic review and meta-analysis aimed to calculate the pooled seroprevalence of immunoglobulin G (IgG) and M (IgM) antibodies against *T. gondii* in solid organ transplant (SOT: kidney, liver, and heart) recipients, adhering to PRISMA guidelines (Moher et al., 2015; Page et al., 2021).

### 2.2 Search strategy

Researchers examined three global databases: Medline/PubMed, Scopus, and the Web of Knowledge up to October 24, 2024. They used Medical Subject Heading (MeSH) terms in various combinations: (“Parasitic Infections” OR “*Toxoplasma gondii*”, OR “Toxoplasmosis”) AND (“Seroprevalence” OR “Epidemiology” OR “Frequency” OR “Occurrence”) AND (“Transplantation” OR “Solid organ” OR “Transplant recipients” OR “Renal transplantation” OR “Liver transplantation” OR “Heart transplantation”) AND (“IgM” OR “IgG”). Additional keywords/manual search were used to encompass relevant studies. Google Scholar was searched for gray literature, and the references of key papers were reviewed. After importing the data, duplicates were automatically eliminated with EndNote X7 software. Notably, two researchers independently evaluated the articles.

### 2.3 Inclusion/exclusion criteria

This global systematic review evaluated cross-sectional and case-control studies conducted in various languages, regions, and timeframes to determine the anti-*T. gondii* IgG and IgM seroprevalence in the post-transplant stage for SOT recipients through serological methods. The review excluded case reports, reviews, commentaries, inaccessible articles, studies involving individuals other than SOT recipients, studies with low quality assessment scores, animal studies, research reporting pre-transplant seroprevalence of *T. gondii*, studies with ambiguous results and classifications, as well as those lacking sample size or prevalence rate data for anti-*T. gondii* antibodies. Of note, pre-transplant studies on *T. gondii* and post-transplant articles containing pre-transplant data were only included to evaluate anti-*T. gondii* IgG serostatus in donors and recipients (D/R).

### 2.4 Quality evaluation

Papers were evaluated for inclusion or exclusion using the Joanna Briggs Institute (JBI) Critical Appraisal Checklist for prevalence studies (Munn et al., 2015). Two researchers extracted essential data from the selected papers, which was then verified by others. This information included the first author's last name, transplanted organ, diagnostic method, quality assessment level, year of publication and implementation, continent, country, WHO classification, total sample size, and number of infected samples.

### 2.5 Meta-analysis

Statistical analyses were conducted using Comprehensive Meta-Analysis (CMA) v3 software, with *P*-values under 0.05 deemed statistically significant. A random-effects model was employed to assess the anti-*T. gondii* IgG and IgM seroprevalence, as well as donor/recipient serostatus in SOT recipients, calculating pooled prevalence and 95 % confidence intervals. Subgroup analysis assessed the anti-*T. gondii* IgG seroprevalence in SOT recipients according to transplanted organ, WHO regions, countries, publication years, continents, and sample sizes. The pooled random effects odds ratio (OR) was calculated from case-control studies to evaluate the association of anti-*T. gondii* seropositivity in SOT recipients. A forest plot displayed the pooled seroprevalence with 95 % confidence intervals. Heterogeneity was quantified with the *I*^*2*^ index, categorizing it as low (under 25 %), moderate (25–50 %), or high (over 50 %). Sensitivity analysis explored changes in the final seroprevalence by excluding single studies/datasets.

### 2.6 Ethics approval

The current study was approved by the Ethics Committee of Qazvin University of Medical Sciences, Qazvin, Iran (approval no. IR. QUMS.REC.1403.300).

## 3 Results

### 3.1 Included articles

**Fig. 1:**
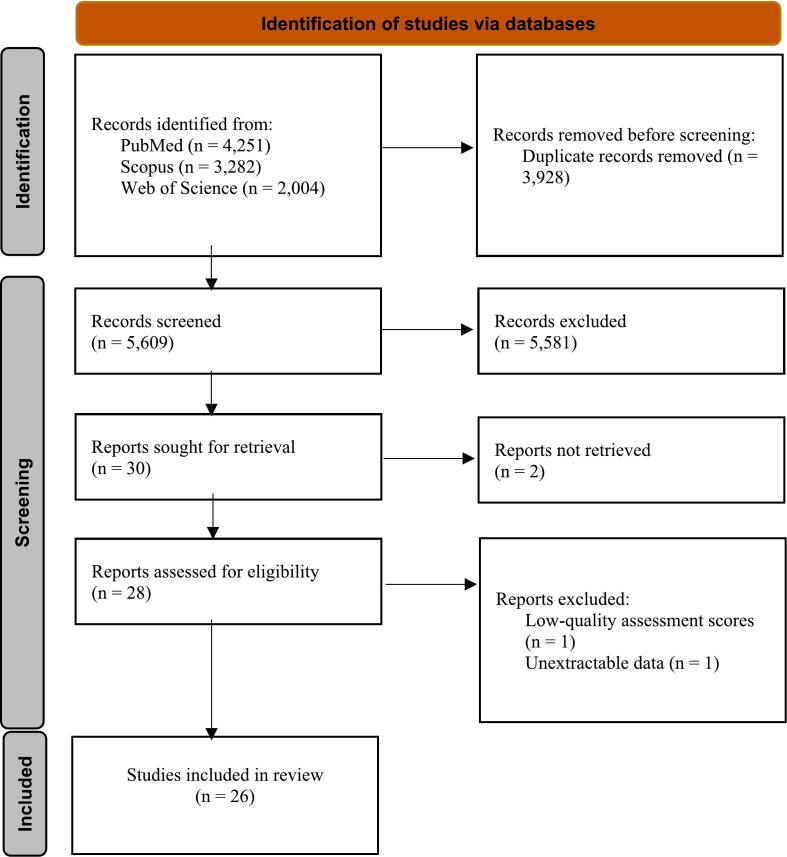
The PRISMA 2020 flow diagram depicting the process of included studies in the present systematic review.

A comprehensive search of four global databases identified 9537 records. After deduplication and review of the 5609 remaining records, 28 articles were selected. Two additional studies were excluded due to JBI quality assessment criteria and unextractable data. Ultimately, 26 relevant studies comprising 29 datasets (Andersson et al., 1992; Arora et al., 2007; Baran et al., 2006; Batista et al., 2011; Caner et al., 2008; Fernàndez-Sabé et al., 2012; Gallino et al., 1996; Galván-Ramírez et al., 2019; Gharavi et al., 2011; Gourishankar et al., 2008; Hamza et al., 2015; Izadi et al., 2013; Mohammed et al., 2021; Orang et al., 2020; Orr et al., 1994; Pinto et al., 2020; Pintos et al., 2023; Raeghi et al., 2011; Rasti et al., 2016; Rostami et al., 2006; Saad et al., 2015; Sluiters et al., 1989; Soltani et al., 2013; Valar et al., 2007; Wreghitt et al., 1987, Wreghitt et al., 1989) met the inclusion criteria for this research (Fig. 1).

### 3.2 Characteristics of the included papers

This review analyzed 29 datasets from 26 studies conducted between 1987 and 2023 to calculate the pooled seroprevalence of anti-*T. gondii* IgG and IgM antibodies in SOT recipients, including 12 studies on renal, 7 on heart, and 5 on liver transplant recipients. Among the 19,391 samples in the case group, there were 10,535 renal, 5718 liver, and 3138 heart transplant recipients. Eight of the 24 examined studies reported anti-*T. gondii* IgG seroprevalence in control groups, totaling 501 renal transplant recipient controls, 316 heart transplant recipient controls, and 63 liver transplant recipient controls. Recipients in the post-transplant stage formed the case groups, while the control groups included donors, healthy participants, and transplantation candidates in the pre-transplant stage. The seroprevalence of anti-*T. gondii* IgM antibodies in SOT recipients was analyzed using available data from 8 of the 29 included datasets. Eight articles with pre-transplant data were used to report donor/recipient IgG serostatus (D+/R–, D−/R–, D−/R+, D+/R+). There were 7 datasets related to Iran, 4 to Brazil, 3 to Spain, 2 to Egypt, and 1 dataset each from Mexico, Netherlands, Norway, Sudan, Sweden, Switzerland, Turkey, and USA, with sample sizes ranging from 30 to 7709 (Table 1). Quality assessment using the JBI checklist showed that 5 studies were of high quality (>6 points) and 21 were of moderate quality (4–6 points) (Supplementary Table 1).

**Table 1:** Data from 21 articles/24 datasets on the anti-*T. gondii* IgG seroprevalence in solid organ transplant recipients.

Author, year	Transplanted organ	Time tested	Country	Cases		Controls		Control population	Method
				Total no.	Infected no. (%)	Total no.	Infected no. (%)		
Sluiters, 1989	Heart	1984–1987	Netherlands	41	28 (68.3)	32	18 (56.2)	Donors	ELISA
Andersson, 1992	Heart	1988–1990	Sweden	75	3 (4)	–	–	–	IIF, ELISA
Gallino, 1996	Heart	1985–1991	Switzerland	121	16 (13)	–	–	–	SMI
Rostami, 2006	Kidney	2003–2004	Iran	551	39 (7.1)	–	–	–	IFA
Baran, 2006	Heart	1989–2004	USA	596	0 (0)	–	–	–	UC
Valar, 2007	Kidney	2001–2005	Brazil	657	1 (0.1)	–	–	–	UC
Arora, 2007	Heart	1994–2005	Norway	288	77 (26.7)	246	47 (19.1)	Donors	ELISA
Caner, 2008	Liver	2005–2006	Turkey	40	27 (67.5)	38	24 (63.1)	Donors	SFD, ISAGA, EIA, PCR
Batista, 2011a	Kidney	2001–2006	Brazil	1046	6 (0.6)	–	–	–	PCR, SER
Batista, 2011b	Liver	2001–2006	Brazil	708	1 (0.1)	–	–	–	PCR, SER
Gharavi, 2011	Kidney	UC	Iran	102	65 (63.7)	102	65 (63.7)	Self-control	ELFA, ELISA
Raeghi, 2011	Kidney	2009–2010	Iran	83	36 (43.3)	–	–	–	ELISA
Fernandez-Sabe, 2012a	Heart	2000–2009	Spain	1979	12 (0.6)	–	–	–	CLIA
Fernandez-Sabe, 2012b	Kidney	2000–2009	Spain	7709	6 (0.8)	–	–	–	CLIA
Fernandez-Sabe, 2012c	Liver	2000–2009	Spain	4872	4 (0.1)	–	–	–	CLIA
Soltani, 2013	Kidney	UC	Iran	100	34 (34)	100	26 (26)	Healthy participants	ELISA, PCR
Izadi, 2013	Kidney	UC	Iran	50	27 (54)	–	–	–	ELISA
Saad, 2015	Liver	2011–2013	Egypt	50	14 (28)	25	0 (0)	Healthy participants	ELISA
Hamza, 2015	Kidney	UC	Egypt	30	21 (70)	30	9 (30)	Healthy participants	EIA
Rasti, 2016	Kidney	2014–2015	Iran	50	26 (52)	120	40 (33.3)	Healthy participants	ELISA
Galvan-Ramirez, 2019	Kidney	2014–2016	Mexico	99	25 (25.2)	99	38 (38.4)	Donors	ELISA, Western blot
Orang, 2020	Heart	2018–2019	Iran	38	0 (0)	38	4 (10.5)	Self-control	ELISA
Mohammed, 2021	Kidney	2019–2020	Sudan	58	21 (36.2)	50	16 (32)	Donors	ELISA
Pintos, 2023	Liver	2008–2018	Brazil	48	28 (58.3)	–	–	–	CLIA

UC: unclear, ELISA: enzyme-linked immunosorbent assay, IIF: indirect immunofluorescence, SMI: semiquantitative microparticle immunoassay, IFA: indirect fluorescent antibody, SFD: Sabin-Feldman dye test, ISAGA: immunosorbent agglutination assay, EIA: immunoenzymatic assay, PCR: polymerase chain reaction, SER: serological detection, ELFA: enzyme-linked flourescence assay, CLIA: chemiluminescence immunoassay.

### 3.3 Pooled seroprevalence of anti-*T. gondii* antibodies in SOT recipients

**Fig. 2:**
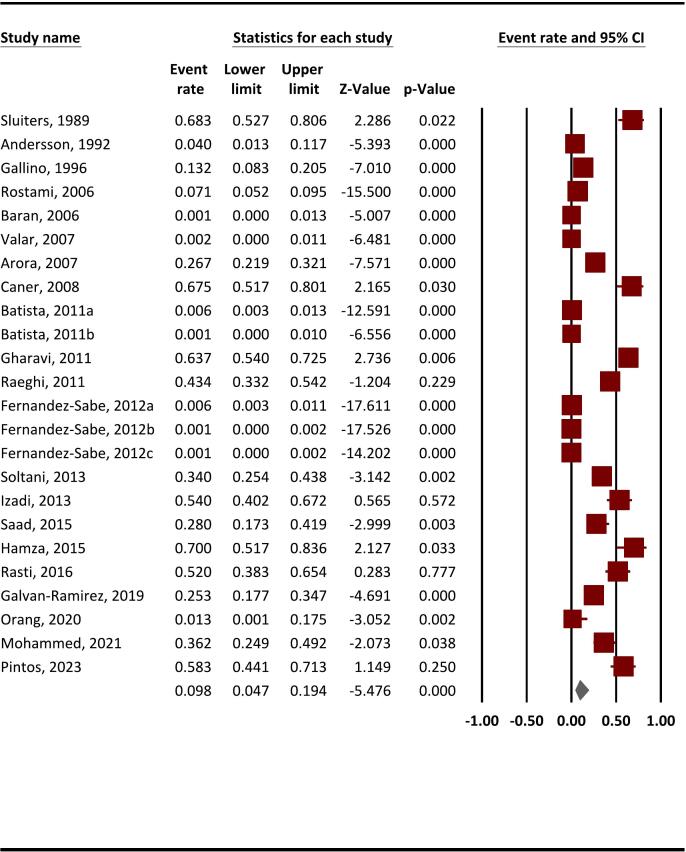
The pooled seroprevalence of anti-*T. gondii* IgG in SOT recipients using a random-effects model and 95 % CIs. * Brown colors indicate the event rate/prevalence reported in each study, while the gray color represents the final weighted prevalence. (For interpretation of the references to color in this figure legend, the reader is referred to the web version of this article.)

**Fig. 3:**
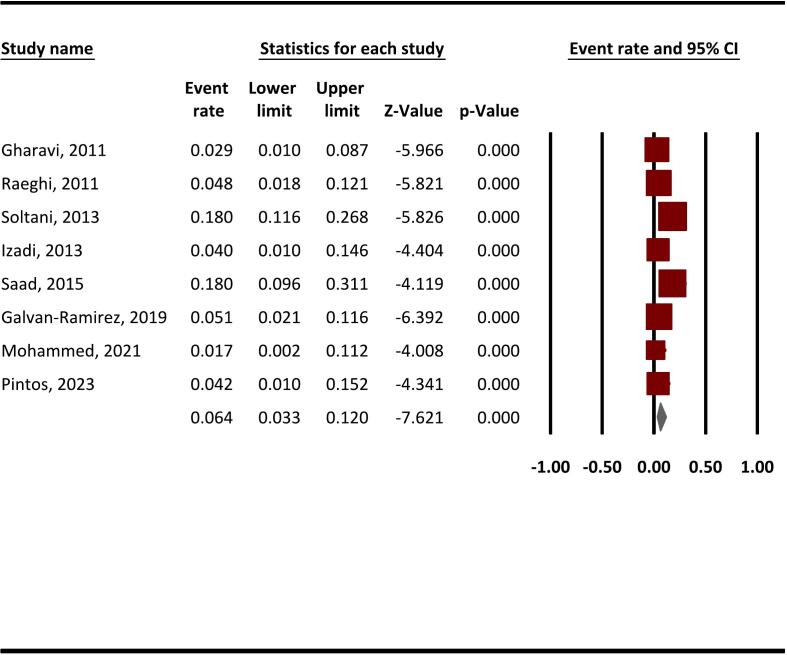
The pooled seroprevalence of anti-*T. gondii* IgM in SOT recipients using a random-effects model and 95 % CIs. * Brown colors indicate the event rate/prevalence reported in each study, while the gray color represents the final weighted prevalence. (For interpretation of the references to color in this figure legend, the reader is referred to the web version of this article.)

The pooled anti-*T. gondii* IgG seroprevalence among SOT recipients was 9.8 % (95 % CI, 4.7–19.4 %) (Fig. 2), while the anti-*T. gondii* IgM seroprevalence was reported at 6.4 % (95 % CI, 3.3–12 %) (Fig. 3). This systematic review and meta-analysis revealed substantial heterogeneity among anti-*T. gondii* IgG reporting studies, evidenced by the statistical analysis (Q = 1061.4, *I*^*2*^ = 97.8 %, *P* < 0.001).

### 3.4 Weighted seroprevalence of anti-*T. gondii* IgG based on transplanted organ

The highest pooled anti-*T. gondii* IgG seroprevalence was found in renal transplant recipients at 14.6 % (95 % CI: 5.5–33.8 %), followed by liver transplant recipients at 6.8 % (95 % CI: 0.5–49.6 %) and heart transplant recipients at 5.4 % (95 % CI: 1.1–22.2 %) (Supplementary Fig. 1 and Table 2).

**Table 2:** Subgroup analysis of anti-*T. gondii* IgG seroprevalence in SOT recipients based on publication year, continent, WHO region, country, sample size, and transplanted organ.

Subgroup variable	Seroprevalence % (95 % CI)	Heterogeneity (Q)	df (Q)	I^2^ (%)	p-value
**Publication year**					
≤2011	8.9 (3.5–20.8)	413.6	11	97.3	p < 0.05
>2011	11.3 (3.2–32.9)	645.8	11	98.3	p < 0.05
**Continent**					
Africa	43.7 (23.2–66.6)	13.1	2	84.8	p < 0.05
Asia	32.2 (14.3–57.4)	191.4	6	96.9	p < 0.05
Europe	4.9 (0.8–24.7)	569.4	7	98.8	p < 0.05
North America	1.9 (0–87.5)	17.5	1	94.3	p < 0.05
South America	1.2 (0–36.6)	163.9	3	98.2	p < 0.05
**WHO region**					
AMR	1.6 (0.2–13)	187.5	5	97.3	p < 0.05
EMR	36.1 (20.4–55.5)	208.1	9	95.7	p < 0.05
EUR	4.9 (0.8–24.7)	569.4	7	98.8	p < 0.05
**Country**					
Brazil	1.2 (0–36.6)	163.9	3	98.2	p < 0.05
Egypt	48.4 (13.9–84.4)	12.4	1	91.9	p < 0.05
Iran	32.2 (14.3–57.4)	191.4	6	96.9	p < 0.05
Mexico	25.3 (17.7–34.7)	0	0	0	p > 0.05
Netherlands	68.3 (52.7–80.6)	0	0	0	p > 0.05
Norway	26.7 (21.9–32.1)	0	0	0	p > 0.05
Spain	0.2 (0–0.7)	22.5	2	91.1	p < 0.05
Sudan	36.2 (24.9–49.2)	0	0	0	p > 0.05
Sweden	4 (1.3–11.7)	0	0	0	p > 0.05
Switzerland	13.2 (8.3–20.5)	0	0	0	p > 0.05
Turkey	67.5 (51.7–80.1)	0	0	0	p > 0.05
USA	0.1 (0–1.3)	0	0	0	p > 0.05
**Sample size**					
≤100	41.3 (30.8–52.6)	88.4	12	86.4	p < 0.05
>100	1.3 (0.3–5.1)	711.1	10	98.6	p < 0.05
**Transplanted organ**					
Heart	5.4 (1.1–22.2)	242.6	6	97.5	p < 0.05
Kidney	14.6 (5.5–33.8)	574.3	11	98.1	p < 0.05
Liver	6.8 (0.5–49.6)	226.9	4	98.2	p < 0.05

### 3.5 Association between SOT recipients and anti-*T. gondii* IgG

**Fig. 4:**
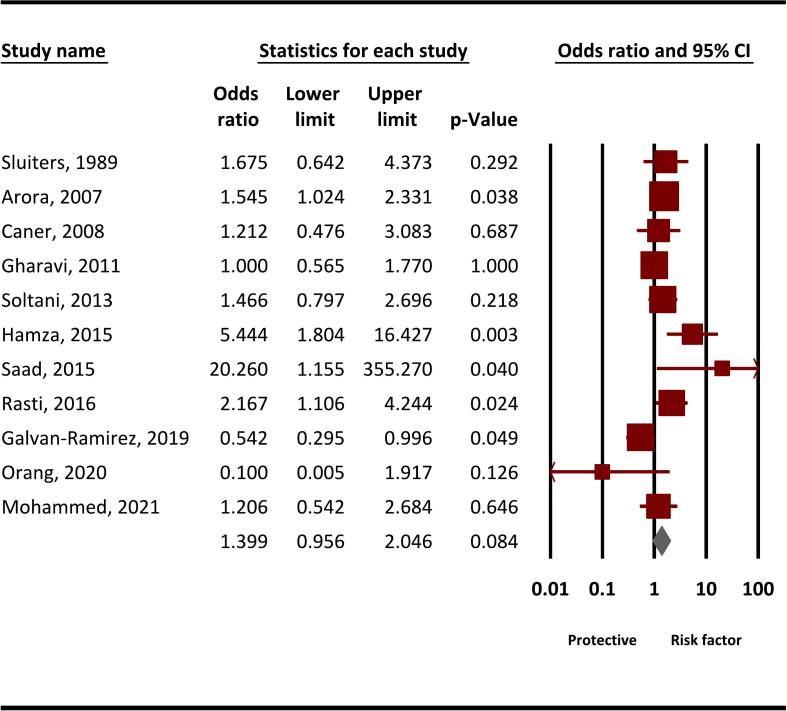
The pooled random effects odds ratio (OR) for infections with *T. gondii* (IgG) in SOT recipients compared to controls. * Brown colors indicate the OR reported in each study, while the gray color represents the final pooled OR. (For interpretation of the references to color in this figure legend, the reader is referred to the web version of this article.)

The pooled random effects OR for infections with *T. gondii* in SOT recipients compared to controls was 1.39 (95 % CI, 0.95–2.04, *P* = 0.08) (Fig. 4).

### 3.6 Pooled anti-*T. gondii* IgG serostatus based on donor/recipient in the initial/pre-transplant serological screening

The highest pooled anti-*T. gondii* IgG serostatus was 50.7 % (95 % CI: 18.2–82.6 %) in the undetermined group, followed by 38 % (95 % CI: 18.6–62.2 %) in the D−/R- group, 15.4 % (95 % CI: 11.1–21 %) in the D−/R+ group, 10.6 % (95 % CI: 5.9–18.5 %) in the D+/R- group, and 9.9 % (95 % CI: 3.9–23.2 %) in the D+/R+ group (Supplementary Fig. 2 and Table 3).

**Table 3:** Serostatus of anti-*T. gondii* IgG based on donor/recipient groups in the initial/pre-transplant serological screening.

Author, year	Total patients	IgG anti-*T. gondii* serostatus (N)					Group based prevalence (%)				
		D+/R–	D−/R–	D−/R+	D+/R+	UN					
Wreghitt, 1987	35	5	13	13	4	–	14.3	37.1	37.1	11.4	–
Wreghitt, 1989	250	21	–	–	–	229	–	8.4	–	–	91.6
Orr, 1994	290	13	211	57	9	–	4.5	72.7	19.6	3.1	–
Caner, 2008	40	3	10	4	21	2	7.5	25	10	52.5	5
Gourishankar, 2008	907	86	664	132	25	–	9.5	73.2	14.5	2.8	–
Fernandez-Sabe, 2012	22	9	1	0	1	11	40.9	4.5	0	4.5	50
Galvan-Ramirez, 2019	99	29	46	14	10	–	29.3	46.6	14.1	10.1	–
Pinto, 2020	409	10	67	39	72	221	2.4	18.4	9.5	17.6	54

**Note:** Patients were grouped according to the basal serology of the binomial donor/recipients. (D + / R +) = seropositive donors/recipients; (D + / R-) = seropositive donors/seronegative recipients; (D- / R +) = seronegative donors /seropositive recipients; and (D- / R -) = seronegative donors and recipients, UN = undetermined serology.

### 3.7 Weighted anti-*T. gondii* IgG seroprevalence in SOT recipients based on examined subgroups

Table 2 displays the anti-*T. gondii* IgG seroprevalence among SOT recipients, organized by publication year, continent, WHO region, country, sample size, and transplanted organ (Supplementary Figs. 1, and 3–7).

### 3.8 Sensitivity analysis

Sensitivity analysis indicated that excluding individual studies or datasets on anti-*T. gondii* IgG seroprevalence in SOT recipients did not significantly affect the pooled seroprevalence (*P* > 0.05) (Supplementary Fig. 8).

## 4 Discussion

In organ transplantation, distinguishing between primary infections in uninfected individuals exposed to *T. gondii* and reactivations of latent infections in seropositive individuals with weakened immune systems is essential (Khurana and Batra, 2016). Serological testing for IgM antibodies is a valuable diagnostic tool, but interpreting the results necessitates caution. In transplant recipients, IgM may indicate a recent infection, although false positives can complicate the diagnosis (Liu et al., 2015; Montoya, 2002; Remington et al., 2004). In contrast, IgG antibodies signify past exposure and, when considered with clinical symptoms and patient history, aid in distinguishing new infections from reactivations (Lappalainen and Hedman, 2004; Robert-Gangneux and Guegan, 2021). Of note, positive results for anti-*T. gondii* IgG antibodies can indicate immunity rather than just exposure to an infection. This means that the presence of these antibodies suggests that the immune system of patients has successfully responded to a previous *T. gondii* infection, potentially providing protection against future infections. Overall, regular monitoring of seroconversion rates and IgM/IgG levels, along with clinical assessments, can inform effective prophylactic and therapeutic strategies for managing *T. gondii* infections in this vulnerable group (Derouin et al., 2008).

In the present study, the pooled seroprevalence of anti-*T. gondii* IgG and IgM among solid organ transplant recipients was 9.8 % and 6.4 %, respectively. The sensitivity analysis indicated no outliers among the included studies/datasets, and excluding individual studies/datasets had no significant impact on the final reported seroprevalence. In a previous systematic review and meta-analysis (Wang et al., 2017), *T. gondii* infection rates were 35.9 % in immunocompromised patients versus 24.7 % in controls (*p* < 0.001), with an OR of 2.24. Specifically, the infection rates were 42.1 % for HIV/AIDS patients compared to 32.0 % in controls (*p* < 0.05), 26.0 % for cancer patients versus 12.1 % in controls (p < 0.001), and 42.1 % for transplant recipients against 34.5 % in controls (*p* > 0.05). The estimated pooled ORs were 1.92 (95 % CI, 1.44–2.55), 2.89 (95 % CI, 2.36–3.55), and 1.51 (95 % CI, 1.16–1.95), respectively. Of note, the previous meta-analysis on *T. gondii* in transplant recipients was based on 6 studies, whereas the current study updates the findings with data from 21 studies and 24 datasets. The rising number of studies indicated a significant drop in *T. gondii* seroprevalence among transplant recipients, from 42.1 % to 9.8 %. Accordingly, the findings from reviews and meta-analyses rely on existing data, which requires periodic updates (Moher et al., 2007; Simmonds et al., 2017). Our findings highlight the significant risk *T. gondii* poses to immunocompromised individuals, particularly transplant recipients with altered immune responses. The findings indicate a need for ongoing surveillance and preventive strategies to reduce the risk of severe *T. gondii* infections, which can cause significant morbidity. Of note, this study's statistical findings, particularly the seroprevalence of anti-*T. gondii* IgM in SOT recipients, are based on limited data and should be interpreted with caution.

The reported seroprevalence of anti-*T. gondii* antibodies in transplant recipients raise critical questions about exposure sources. Investigating environmental factors, dietary habits, and the prevalence of infected cats, major reservoirs of *T. gondii*, is essential for developing effective patient care guidelines. Contact with cats and exposure to soil have been linked to increased seroprevalence, suggesting that environmental exposure plays a significant role in transmission of *T. gondii* infections (Wang et al., 2020). Moreover, consumption of undercooked or raw meat has been associated with higher seroprevalence of *T. gondii*, highlighting the importance of dietary considerations in preventing infection (Wilking et al., 2016). Given the risks of seropositivity, particularly with the presence of IgM antibodies suggesting recent infection, clinicians must remain vigilant for signs of *T. gondii* infection. Routine screening and prompt intervention during seroconversion are essential for improving outcomes in these vulnerable individuals. In transplant recipients, toxoplasmosis can result from donor-transmitted infection, reactivation of latent infection, or de novo infection. Clinical manifestations often occur within the first three months' post-transplant and may present as encephalitis, pneumonitis, chorioretinitis, meningitis, or disseminated toxoplasmosis with multi-organ involvement (Khurana and Batra, 2016).

Analysis by year of publication indicated that the highest seroprevalence of anti-*T. gondii* IgG antibodies in SOT recipients occurred after 2011 (11.3 %, 95 % CI: 3.2–32.9). SOT recipients in Africa and the EMR WHO region exhibited higher seroprevalence, with the Netherlands (68.3 %; 95 % CI: 52.7–80.6 %), Turkey (67.5 %; 95 % CI: 51.7–80.1 %), Egypt (48.4 %; 95 % CI: 13.9–84.4 %), Sudan (36.2 %; 95 % CI: 24.9–49.2 %), and Iran (32.2 %; 95 % CI: 14.3–57.4 %) showing the highest rates among the 12 countries studied. The study also found a direct correlation between larger sample sizes and lower seroprevalence of anti-*T. gondii* IgG antibodies in SOT recipients, highlighting the need for sufficient studies/sample sizes in epidemiological/meta-analysis research to accurately reflect disease or infection prevalence. This study found the highest pooled anti-*T. gondii* IgG seroprevalence in renal transplant recipients, followed by liver and heart transplant recipients. Despite differences in the number of studies, patients, and positive cases across groups, the lower seroprevalence in heart and liver transplant recipients compared to renal transplant recipients may indicate variations in immunosuppression regimens or exposure risks. These findings highlight the need for tailored preventative measures and further research to comprehend *T. gondii*'s impact on the transplant population.

The pooled random effects odds ratio for *T. gondii* infections in solid organ transplant recipients compared to controls was 1.39 (95 % CI, 0.95–2.04, *P* = 0.08). This indicates a potential but not statistically significant increase in the risk of *T. gondii* infections among SOT recipients relative to controls. The observed odds ratio suggests that SOT recipients may have a 39 % higher likelihood of infection. Further investigation is warranted to assess the underlying factors contributing to this trend, including immunosuppressive therapies, the type of organ transplanted, and patient demographics. A larger sample size and more rigorous control for confounding variables may be necessary in future studies to elucidate the true impact of *T. gondii* on this vulnerable population.

The evaluation of anti-*T. gondii* IgG serostatus among donor/recipient groups showed that the pooled prevalence of D−/R- group was 38 %, higher than other groups (D−/R+, D+/R-, and D+/R+). However, the D+/R- group, with a pooled prevalence of 10.6 %, remains a significant risk group that should not be overlooked. Transplanting an organ from a *T. gondii*-positive donor (D+) to a seronegative recipient (R−) carries a high infection risk, especially for immunocompromised patients. *T. gondii* infection can lead to serious health complications, such as encephalitis and systemic infections (Butani and Tancredi, 2024; Doesch et al., 2010; Snyder et al., 1988). For transplant recipients with suppressed immune systems, these infections can have severe, potentially life-threatening consequences (Foroutan-Rad et al., 2016). Understanding D+/R− status aids in informed decision-making for organ allocation and recipient selection, enabling effective preventative measures like donor screening and recipient matching (Kanno et al., 2024; Wesołowski et al., 2023). Overall, aligning donor and recipient serostatus can enhance transplant outcomes, reduce complications, and improve overall transplantation success rates. Of note, these mentioned results are based on limited number of studies and confined seroprevalence data, necessitating cautious interpretation.

This study provided comprehensive and current information on the seroprevalence of anti-*T. gondii* antibodies in SOT recipients and used appropriate statistical methods for data analysis, but it had several limitations. Key issues included a small sample size in some studies, limited geographic diversity, reliance on single studies for specific analyses, inadequate data on variables such as age, gender, and immune status, absence of antigen-based confirmatory tests, and dependence on antibody tests that may produce false-positive results.

## 5 Conclusion

This systematic review and meta-analysis revealed a 9.8 % pooled seropositivity for anti-*T. gondii* IgG among SOT recipients, with notable variations by region and organ type. Renal transplant recipients exhibited a higher risk of *T. gondii* exposure. The 6.4 % seroprevalence of anti-*T. gondii* IgM indicates active infections, emphasizing the need for careful post-transplant monitoring. An OR of 1.39 signifies an increased risk, underscoring the necessity of thorough donor screening and effective management of recipients. Variations in seroprevalence linked to serostatus highlight the complexity of *T. gondii* transmission, underscoring the need for tailored prevention strategies. These findings call for increased awareness and preventive measures to improve post-transplant outcomes and patient safety. Further research is crucial to understand these disparities and develop management protocols for *T. gondii* in transplant recipients.

## CRediT authorship contribution statement

**Mina Mamizadeh:** Writing – review & editing, Methodology. **Farajolah Maleki:** Writing – original draft, Methodology, Investigation. **Mohammad Reza Mohammadi:** Writing – original draft, Methodology, Investigation. **Laya Shamsi:** Methodology, Investigation. **Ali Asghari:** Writing – review & editing, Writing – original draft, Supervision, Methodology, Investigation, Conceptualization. **Ali Pouryousef:** Writing – review & editing, Methodology, Investigation.

## Declaration of competing interest

The authors declare no potential conflicts of interest with respect to the research, authorship, and/or publication of this article.

## Appendix A Supplementary data

Supplementary material 1 Image 1

Supplementary material 2 Image 2

Supplementary material 3 Image 3

Supplementary material 4 Image 4

Supplementary material 5 Image 5

Supplementary material 6 Image 6

Supplementary material 7 Image 7

Supplementary material 8 Image 8

Supplementary material 9 Image 9
