# Supplementary material for: Seroprevalence and risk factors for Toxoplasma gondii infection in solid organ transplant patients: A global systematic review and meta-analysis
Source: Parasite Epidemiol Control. 2025 Mar 7;29:e00421. doi: 10.1016/j.parepi.2025.e00421 (PMC11932682; doi:10.1016/j.parepi.2025.e00421)
Supplement: Supplementary file 2 — Supplementary material 2 [file mmc2.docx]

**Supplementary Fig. 2.** The pooled anti-*T. gondii* IgG serostatus based on donor/recipient in the initial/pre-transplant serological screening, using a random-effects model and 95% CIs.
