# Supplementary material for: Seroprevalence and risk factors for Toxoplasma gondii infection in solid organ transplant patients: A global systematic review and meta-analysis
Source: Parasite Epidemiol Control. 2025 Mar 7;29:e00421. doi: 10.1016/j.parepi.2025.e00421 (PMC11932682; doi:10.1016/j.parepi.2025.e00421)
Supplement: Supplementary file 8 — Supplementary material 8 [file mmc8.docx]

**Supplementary Fig. 8.** Sensitivity analysis showing the impact of excluding individual studies/datasets on final anti-*T. gondii* IgG seroprevalence in SOT recipients, based on a random-effects model with 95% confidence intervals.
