# Supplementary material for: Seroprevalence and risk factors for Toxoplasma gondii infection in solid organ transplant patients: A global systematic review and meta-analysis
Source: Parasite Epidemiol Control. 2025 Mar 7;29:e00421. doi: 10.1016/j.parepi.2025.e00421 (PMC11932682; doi:10.1016/j.parepi.2025.e00421)
Supplement: Supplementary file 9 — Supplementary material 9 [file mmc9.docx]

**Table1. JBI critical appraisal checklist applied for included studies**

| **Cross-sectional** | | | | | | | | | | | | |  |  |  |
| --- | --- | --- | --- | --- | --- | --- | --- | --- | --- | --- | --- | --- | --- | --- | --- |
| **Author Name/Year** | **Q1** | **Q2** | **Q3** | **Q4** | **Q5** | **Q6** | **Q7** | **Q8** | **Overall Quality** | | | |  |  |  |
| Andersson, 1992 | Yes | No | Yes | UN | Yes | UN | Yes | Yes | 5 | | | |  |  |  |
| Gallino, 1996 | Yes | Yes | No | Yes | NO | UN | Yes | Yes | 5 | | | |  |  |  |
| Rostami, 2006 | UN | Yes | Yes | Yes | Yes | UN | UN | Yes | 5 | | | |  |  |  |
| Baran, 2006 | Yes | Yes | No | Yes | Yes | UN | Yes | Yes | 6 | | | |  |  |  |
| Valar, 2007 | Yes | Yes | Yes | Yes | No | Yes | Yes | No | 6 | | | |  |  |  |
| Batista, 2011 | Yes | Yes | No | Yes | Yes | No | Yes | Yes | 6 | | | |  |  |  |
| Raeghi, 2011 | Yes | UN | Yes | Yes | Yes | Yes | No | Yes | 6 | | | |  |  |  |
| Fernandez-Sabe, 2012 | Yes | No | Yes | NO | Yes | UN | Yes | Yes | 5 | | | |  |  |  |
| Izadi, 2013 | Yes | Yes | No | Yes | Yes | UN | Yes | Yes | 6 | | | |  |  |  |
| Pintos, 2023 | UN | Yes | Yes | Yes | Yes | UN | UN | Yes | 5 | | | |  |  |  |
| Wreghitt, 1987 | No | Yes | No | Yes | Yes | UN | Yes | NO | 4 | | | |  |  |  |
| Wreghitt, 1989 | Yes | No | Yes | NO | Yes | UN | NO | Yes | 4 | | | |  |  |  |
| Orr, 1994 | NO | Yes | No | Yes | Yes | UN | Yes | Yes | 5 | | | |  |  |  |
| Gourishankar, 2008 | UN | Yes | Yes | Yes | Yes | UN | UN | Yes | 5 | | | |  |  |  |
| Pinto, 2020 | Yes | Yes | UN | Yes | Yes | Yes | Yes | Yes | 7 | | | |  |  |  |
| **Case-control** | | | | | | | | | | | | |  |  |  |
| **Author Name/Year** | **Q1** | **Q2** | **Q3** | **Q4** | **Q5** | **Q6** | **Q7** | **Q8** | **Q9** | **Q10** | **Overall Quality** | |  |  |  |
| Sluiters, 1989 | Yes | UN | Yes | No | UN | Yes | UN | Yes | UN | Yes | 5 | |  |  |  |
| Arora, 2007 | Yes | Yes | UN | Yes | Yes | NO | UN | Yes | UN | Yes | 6 | |  |  |  |
| Caner, 2008 | UN | Yes | Yes | UN | No | Yes | Yes | Yes | Yes | Yes | 7 | |  |  |  |
| Gharavi, 2011 | Yes | NO | UN | Yes | Yes | UN | UN | NO | Yes | Yes | 5 | |  |  |  |
| Soltani, 2013 | Yes | Yes | UN | Yes | Yes | Yes | No | Yes | UN | Yes | 7 | |  |  |  |
| Saad, 2015 | Yes | Yes | No | Yes | Yes | NO | Yes | Yes | Yes | UN | 7 | |  |  |  |
| Hamza, 2015 | Yes | Yes | UN | Yes | Yes | Yes | UN | Yes | UN | Yes | 7 | |  |  |  |
| Rasti, 2016 | Yes | Yes | NO | UN | NO | Yes | Yes | No | Yes | Yes | 6 | |  |  |  |
| Galvan‐Ramirez, 2019 | Yes | NO | UN | Yes | Yes | UN | UN | Yes | NO | Yes | 5 | |  |  |  |
| Orang, 2020 | NO | Yes | UN | Yes | Yes | Yes | UN | Yes | UN | Yes | 6 | |  |  |  |
| Mohammed, 2021 | UN | Yes | Yes | UN | Yes | Yes | Yes | No | Yes | NO | 6 | |  |  |  |
| Cohort | | | | | | | | | | | | |  |  |  |
| Author Name/Year | Q1 | Q2 | Q3 | Q4 | Q5 | Q6 | Q7 | Q8 | Q9 | Q10 | Q11 | Overall Quality |  |  |  |
|  |  |  |  |  |  |  |  |  |  |  |  |  |  |  |  |
| **Cross-sectional questions:**  Q1. Were the criteria for inclusion in the sample clearly defined?  Q2. Were the study subjects and the setting described in detail??  Q3. Was exposure measured in a valid and reliable way?  Q4. Were objective, standard criteria used for measurement of the condition?  Q5. Were confounding factors identified?  Q6. Were strategies to deal with confounding factors stated?  Q7. Were the outcomes measured in a valid and reliable way?  Q8. Was appropriate statistical analysis used?  **Case – control design questions:**  Q1. Were the groups comparable other than the presence of disease in cases or the absence of disease in controls?  Q2. Were cases and controls matched appropriately?  Q3. Were the same criteria used for identification of cases and controls?  Q4. Was exposure measured in a standard, valid and reliable way?  Q5. Was exposure measured in the same way for cases and controls?  Q6. Were confounding factors identified?  Q7. Were strategies to deal with confounding factors stated?  Q8. Were outcomes assessed in a standard, valid and reliable way for cases and controls?  Q9. Was the exposure period of interest long enough to be meaningful?  Q10. Was appropriate statistical analysis used? | | | | | | | | | | | | |  |  |  |
